# Designing a Personalized Health Dashboard: Interdisciplinary and Participatory Approach

**DOI:** 10.2196/24061

**Published:** 2021-02-09

**Authors:** Miriam Weijers, Caroline Bastiaenen, Frans Feron, Kay Schröder

**Affiliations:** 1 Department of Preventive Child Health Care Municipal Health Services, Southern Limburg Heerlen Netherlands; 2 Department of Social Medicine Faculty of Health, Medicine and Life Sciences Care and Public Health Research Institute, Maastricht University Maastricht Netherlands; 3 Department of Epidemiology Faculty of Health, Medicine and Life Sciences Care and Public Health Research Institute, Maastricht University Maastricht Netherlands; 4 Data Visualization Zuyd University of Applied Sciences Heerlen Netherlands

**Keywords:** visualization design model, dashboard, evaluation, personalized health care, International Classification of Functioning, Disability and Health (ICF), patient access to records, human–computer interaction, health information visualization

## Abstract

**Background:**

Within the Dutch Child Health Care (CHC), an online tool (360° CHILD-profile) is designed to enhance prevention and transformation toward personalized health care. From a personalized preventive perspective, it is of fundamental importance to timely identify children with emerging health problems interrelated to multiple health determinants. While digitalization of children’s health data is now realized, the accessibility of data remains a major challenge for CHC professionals, let alone for parents/youth. Therefore, the idea was initiated from CHC practice to develop a novel approach to make relevant information accessible at a glance.

**Objective:**

This paper describes the stepwise development of a dashboard, as an example of using a design model to achieve visualization of a comprehensive overview of theoretically structured health data.

**Methods:**

Developmental process is based on the nested design model with involvement of relevant stakeholders in a real-life context. This model considers immediate upstream validation within 4 cascading design levels: Domain Problem and Data Characterization, Operation and Data Type Abstraction, Visual Encoding and Interaction Design, and Algorithm Design. This model also includes impact-oriented downstream validation, which can be initiated after delivering the prototype.

**Results:**

A comprehensible 360° CHILD-profile is developed: an online accessible visualization of CHC data based on the theoretical concept of the International Classification of Functioning, Disability and Health. This dashboard provides caregivers and parents/youth with a holistic view on children’s health and “entry points” for preventive, individualized health plans.

**Conclusions:**

Describing this developmental process offers guidance on how to utilize the nested design model within a health care context.

## Introduction

The Dutch Preventive Child Health Care (CHC), as part of public health, monitors children’s health and their continuum of development with focus on protecting and promoting health and providing context for optimal development. This implicates preventing disease progression at early stages of a “growing into deficit,” when symptoms do not cluster to a diagnosis or are even absent yet [1]. It is not easy to timely redirect these complex dynamics underlying health. The Bio-Psycho-Social perspective on health (BPS) displays the complexity by conceptualizing health as a result of lifelong, multidimensional interactions between individual (biological–genetic) characteristics and contextual factors [2].

This makes prevention challenging, but it is crucial to effectively address current burden of chronic diseases [3]. It is even a prerequisite that the current health care system, which is mostly reactive (ie, treatment after a diagnosis), transforms toward personalized health care (PHC) [4]. According to Snyderman, PHC includes the concepts prevention, prediction, personalization, and participation and to fully adopt these concepts within practice, the availability of qualitative, holistic health information is required [5,6].

The preventive CHC offers a unique platform to adopt these PHC concepts, as CHC (from birth on) digitally registers a broad spectrum of information about interrelated health determinants in child and environment [1,7]. Yet, the holistic health information, stored in the CHC’s electronic medical dossier (EMD), is insufficiently accessible to effectively perform PHC. The actual data flow is time-consuming due to an inconsistent, nontheoretical structure of the EMD [8-10]. This challenges CHC professionals to gain clear overview of relevant CHC data within the limited timeframe available during consultations with parents and other caregivers. Consequently, CHC professionals are hindered in obtaining integral insight into the interrelated health determinants in child and environment, let alone parents and youth.

To acquire better overview of meaningful data, indispensable for interpretation of holistic health information, the idea was initiated from CHC practice to develop a novel approach for summarizing health data about child and its environment in 1 image [2,11]. Visualization design offers efficient opportunities to make holistic health information accessible at a glance and conform to the relevant theoretical perspective [12,13].

The initial idea was first converted into rough drafts of representation of CHC health information. To enable generation of informal development ideas, the researchers presented first drafts to parents, youth, and CHC professionals and asked for their reaction. Stakeholder’s feedback on these first drafts during interviews (parents) and focus group meetings (professionals) was positive concerning comprehensibility, relevance, acceptability, and feasibility. A pilot study of an early-on version of the 360° CHILD-profile also showed positive results regarding reliability and validity, when used by CHC medical doctors to assess child functioning [14].

The 360° CHILD-profile seemed a promising new tool, but further development was needed to deliver a suitable and functional dashboard, ready to be introduced to CHC practice. To realize meaningful visualization of complex health information with sufficient user satisfaction and essential performance in practice, it is important that such a developmental process is guided by appropriate design models.

The main aim of this paper is to offer guidance on how to utilize a design model to visualize and structure health data in a health care context with a heterogeneous target group. As an example, we describe the systematic development and immediate validation (as far as possible) of a comprehensible 360° CHILD-profile: an online accessible visualization of CHC data. The ultimate goal of this multifunctional tool for preventive CHC practice is to visualize the coherence between health domains in a way that it guides analytic thought processes of both care providers and parents/youth in line with BPS perspective on health and PHC. This paper focusses on describing the overall development process of a visualization tool to offer a clear, representative content generalizable to various subfields and disciplines in health care.

## Methods

### Process Development and Prototype

**Figure 1:**
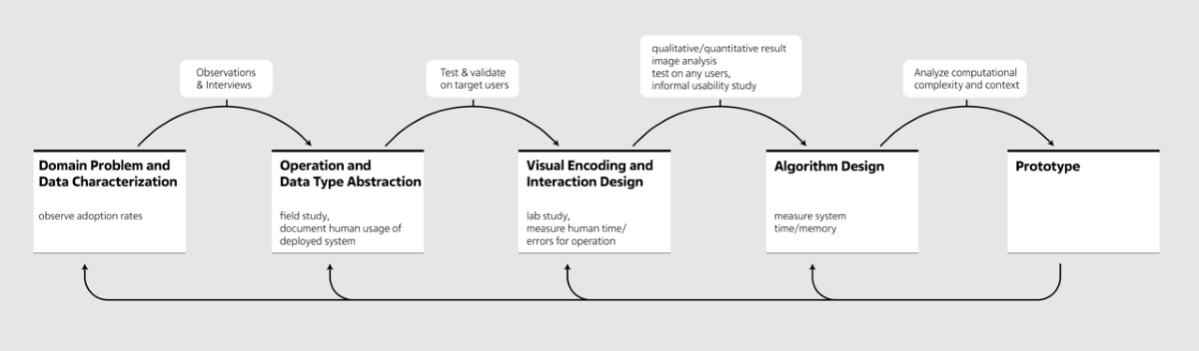
The nested design model, adapted from Munzner [15]. The upper part shows the relevant stages of upstream validation, while the bottom part shows the different dimensions of downstream validation.

The developmental process of the 360° CHILD-profile is based on a nested design model, adapted from Munzner [15] (Figure 1). This model describes different levels of design that are structured within 4 cascading levels that consider an immediate upstream validation (toward delivering a suitable prototype of the dashboard) as well as impact-oriented downstream validation of the prototype (toward the effective performance of the dashboard in daily CHC practice).

The prototype of the CHILD-profile is developed within a user-centered design process [16] and relevant stakeholders were involved during every level of design. For each design level, new participants were recruited. During this project, we collaborated in an interdisciplinary expert group including CHC professionals and researchers with expertise on CHC context, epidemiology, human–computer interaction, and information visualization in health care. This approach, combining expertise from the medical field with expertise on information visualization, is rather new but particularly useful in this health care context to increase the likelihood of the intended health outcome [17].

The Medical Ethics Committee of the Maastricht University Medical Centre approved this design process (METC azM/UM 17-4-083).

Before starting the first level of the nested design model, a literature research was performed with focus on theoretical models for health and background of the Dutch preventive CHC to identify the information needed for each design level.

### Domain Problem and Data Characterization

On the first level, it was of vital importance to bridge the information asymmetry between relevant stakeholders, researchers, and designers to get a common understanding of user, domain, and task [18]. To achieve this while considering the privacy of the users, we first conducted role games, in which CHC consultations were re-enacted in a real-life situation with key stakeholders (CHC professionals, parents, and youth). A schematic approach (summative representation of data to make sense of complex, nuanced information and enable team-based analysis) was used to observe and interpret interpersonal interactions [19]. In the second step, interviews with participants of the role games as well as other CHC professionals were carried out to get a deeper understanding of the process and related requirements from the perspective of individual stakeholders. Role games and interviews were audio recorded. Recordings were summarized and, after discussion by a team of researchers, relevant findings were listed.

Finally, the resulting conclusions about user’s perspectives were immediately validated in real-life by observing consultation hours. During the observations, field notes were taken. Based on the information collected within the previous steps, personas and empathy maps were created to visualize users’ characteristics, goals, and skills, to become more aware of their real needs and to help the research group align on a deep understanding of end users [20,21].

In parallel, the relevant domain knowledge was discussed and summarized with all involved stakeholders to ensure that the involved researchers/designers share a common understanding of the underlying concepts and mechanisms. Furthermore, related work in the field and visual artifacts were discussed.

In summary, all our findings formed the domain-specific basis for the other levels (Figure 1).

### Operation and Data Type Abstraction

The focus of the second level was on mapping the underlying data in a more abstract description of operations, data types, and structure to form the input required for the visual encoding stage.

Different theoretical frameworks were explored to choose the most relevant framework for prioritizing and ordering data. The International Classification of Functioning, Disability and Health: Children and Youth version (ICF-CY) framework appeared to be the most appropriate to comprehensively and accurately describe individual health situations [22]. The classification systems ICD-11 (International Classification of Diseases, 11th revision) and DSM-5 (Diagnostic and Statistical Manual of Mental Disorders, version 5), commonly used in health care, were also considered. However, these frameworks do not fit preventive CHC because they are based on a biomedical model of health and focus on diseases and diagnosis and not on prevention [23,24]. The ICF-CY framework was chosen because it represents the broad BPS perspective on health and adequately fits the preventive CHC. The ICF-CY framework enables to display the broad variety of information on characteristics of a child and its environment, collected by CHC. Strengths and protective factors, inevitable for protection and promotion of health and prevention of diseases, are included in the ICF-CY framework. Next, symptoms, diseases, and determinants that challenge health can be presented. And, last but not least, information is formulated in concrete and neutral, if not positive, terms with little to no valuation. The ICF-CY structure was customized to integrate it into a profile that fits CHC practice and theoretical background.

During 2 review group meetings, the 360° CHILD-profile was presented and profile’s content, terminology, and ordering were discussed with experienced CHC professionals. During the review meetings, field notes were taken and summarized and discussed to reach consensus.

For immediate validation, a static, adapted, early-on version of the 360° CHILD-profile was presented to parents and youth and semistructured interviews were performed to gain insight into user experience (comprehensibility and usability), requirements, and coverage of meaningful topics. Audio recordings of the interviews were transcribed, field notes were taken, and data were analyzed according to previous steps.

Findings were discussed in brainstorming sessions with the research team to verify coherence with scientific and practical purpose of the profile and generate developmental ideas. The resulting findings were not just limited to the data structure and detailed task definitions, but also included meaningful ordering of the information.

### Visual Encoding and Interaction Design

The first 2 levels of design (Domain Problem Characterization and Operation and Data Type Abstraction) formed the primary input for the visual encoding and interaction design on a content level. The development of the formal level was based on 2 additional pillars: the consideration of international standards of human–computer interaction for information representation (ISO 9241-12) [25] as well as theoretical aspects of design based on prior research in this field [26,27] and the systematic integration of users within iterative validation and optimization cycles.

In early stages of the design process, prior findings were integrated into low-fidelity prototypes to conceptually visualize the relevant CHC data and test them with users.

**Figure 2:**
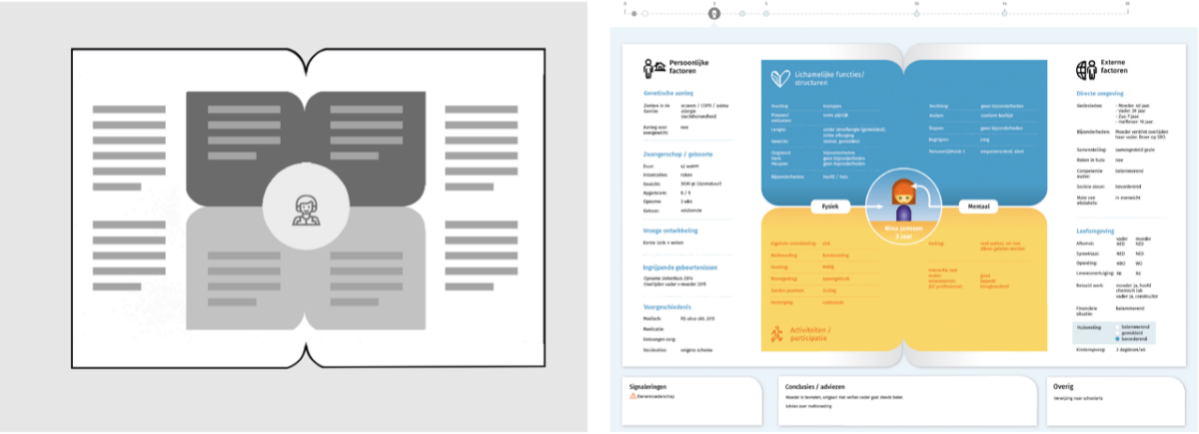
The figures illustrate relevant steps of our iterative design process. The first phase resulted in a global composition design that accurately reflects the content structure (grid layout on the left). In a next step, the design was optimized through additional dimensions such as color, visual language elements such as icons, and illustrations (prototype on the right).

A clear and accessible information structure appeared to be of vital importance to address requirements of the given scenario and a clear visual structure plays a major role in reducing the cognitive load and controlling the perceptual ordering [28]. Therefore, the design was developed based on a sectional grid system and information was structured into areas. The key areas were placed within the center (Figure 2, left) and to facilitate the understanding, key concepts were illustrated through icons in combination with text [29].

The resulting sketch was operationalized into a digital prototype, suitable for informal, qualitative tests with relevant stakeholders (CHC professionals and parents). Participants performed tasks within representative scenarios (to prepare for or to reflect upon a CHC consultation) while considering the profile in all its bearings. Participants were asked to express their first impression on the profile, line out the profile’s structure, seek and interpret specific information, and indicate comprehensibility of information. A researcher guided and facilitated the participants during the sessions. To gain feedback on accessibility, comprehensibility, and usability of the 360° CHILD-profile for each user group, a “think aloud” procedure was conducted [30]. A second researcher observed the session and conducted interviews with the stakeholders. Audio recordings of the interviews were summarized, field notes were taken, and data were analyzed according to previous steps.

For this visualization in accordance with the ICF-CY framework, it is crucial that it stimulates viewers to take into account all domains and choose a routing from central (child) toward outside (environmental factors). Therefore, a gaze tracking evaluation was applied (Tobii X1 Light eye tracker 30 Hz) to gain indirect feedback on what parts of the profile the stakeholders looked at and in which order. Results were discussed in the research team meetings and eventually processed to deliver a digital application of the final version of the online accessible CHILD-profile.

### Algorithm Design

The prototype was developed as a web application based on JavaScript and embedded within an HTML website to ensure an integration into real-life scenarios. Data parsing and mapping were realized through Data Driven Documents (D3) Version 4, while the interactions were implemented using jQuery, JavaScript, and CSS.

The technical implementation was immediately validated in 2 ways: the application was tested by analyzing computational complexity and content and optimized with Chrome DevTools (developer tools) as well as user tests with representative data samples.

### Prototype and Downstream Validation

Downstream validation at the level of algorithm design and visual encoding and interaction design was immediately tackled within the described levels: application test and user tests (see the “Algorithm Design” section) and informal qualitative tests (see the “Visual Encoding and Interaction Design” section).

Downstream validation of the delivered prototype at the level of operation and data type abstraction is beyond the scope of this article. For this dimension of validation, a field study is planned to evaluate CHILD-profile’s feasibility (usability and potential effectiveness) and feasibility of performing a randomized controlled trial (RCT) within the preventive CHC context [31]. This feasibility RCT aims at generating knowledge on how to build follow-up studies directed toward downstream validation at the level of domain problem and data characterization.

## Results

### Domain Problem and Data Characterization

Within more age categories, a total of 3 role games were performed and all involved CHC professionals (nurses or medical doctors or both), parents, and in one case youth (age >12) were interviewed. For field validation, for 2 days, CHC consultation hours were observed in more age categories.

Observations and interviews showed that CHC nurses mainly perform regular, protocoled tasks, and CHC medical doctors mostly explore indicated concerns and problems more in depth. An example of schematic description of a professional within the CHC context (an integration of empathy map and persona) is provided in Multimedia Appendix 1. One of the key challenges we could identify within this level was that the visual structure and interaction design of the current EMD did not sufficiently address the informational needs of the target group. During the interviews and observations, it became apparent that this leads to fundamental problems to fulfill several tasks in the given time due to an ineffective information and interaction structure. Both CHC nurses and medical doctors noted that data registration in the EMD is time-consuming and that they are hindered in quickly referring to registered data and gaining clear overview of health information. Discussion between researchers on visual artifacts revealed the lack of overview and theoretical ordering of data within the EMD. During consultations, CHC professionals pursue active participation of parents and youth but they indicated the need for visual support for communicating health information with parents. Parents indicated the importance of being able to decide for themselves and feeling free to make their own choices during the upbringing of their child. Related work regarding visual support on health communication and revealing parent’s perspectives did not provide a holistic and structured display (in accordance with the ICF-CY framework) of the large and complex electronic CHC data sets [32].

Together with users we developed a description of formal requirements for the CHILD-profile to be designed. The design of the CHILD-profile should be:

- lively and user-friendly with neutral, serene, and warm (fear reducing) appearance to create a positive experience;
- targeted at supporting communication between CHC professionals and parents/youth and providing comprehensible and accurate overview of health determinants in child and its environment.

The pursued ordering effects were allocating the child in a central position, visualizing the coherence between the multiple features in child and context (in accordance with the ICF-CY framework), and making complex health information tangible. Technical requirements for the application were suitability for desktop (for visual support during consultations) and online accessibility but it should also be printable as PDF (A4 format, to be used during house visits).

### Operation and Data Type Abstraction

Content and data ordering for the CHILD-profile were based on the ICF-CY framework, resulting in 4 domains: “Body structures and functions,” “Activities and participation,” “Environment,” and “Personal factors.” The specific content of each domain was customized to the specific Dutch CHC practice and is in accordance with CHC’s professional framework and “toolbox” [33,34]. During 2 review group meetings, the CHC professionals (2 nurses and 2 medical doctors) indicated that the clear overview, ordering of data, and the use of colors were an improvement on accessibility in comparison to the currently used EMD. They proposed even more emphasis on neutral (nuanced) and positive formulations. Second, as not all items are equally relevant during the continuum from age 0 to 18, the review group prioritized specific content for the different age groups (0-15 months, 15 months to 4 years, 4-9 years, 9-12 years, and 12-18 years). Consensus was reached on expert agreement and adaptations were made on prioritization per age category and more positive terminology of data.

### Visual Encoding and Interaction Design

The visualization was designed while taking into account the CHC context, user experiences of prototypes, user’s desires, formal and technical requirements, and the indicated options for improvement of this data visualization.

The qualitative tests of prototypes (on average 30 minute sessions) showed that both target groups could handle the prototype well and performed most of the given tasks correctly (CHC professionals: 7 tasks of 9; parents: 6 tasks of 9). Most participants could link different domains in which health facilitators and barriers are described. Stakeholders feedback on the prototypes included mostly positive remarks such as “nice to build up information during lifetime”, “nice that not only risks factors but also protective factors are included in the overview” and “good to see coherence between health determinants”. However, some parents mentioned the following remarks: “it is a lot of data, in the beginning it is hard to know where to start”, “it is important that formulations are clear”. Participants indicated that in some CHILD-profiles they missed specific information about the child and that it is important to know where the data come from. As participants mentioned the importance of showing a timeline and a separate conclusion section to highlight critical information regarding the last consultation, these elements were incorporated in the final version of the CHILD-profile. Gaze-tracker output showed that all participants explored the profile by starting at the center (child icon/image) and clearly distinguished the middle planes from outer columns. Almost all domain titles were noticed except for “Activities & Participation” and participating professionals often paid more attention to the “conclusion/advice” section than parents.

### Algorithm Design

This algorithm design phase resulted in an application which automatically transfers CHC health data registered in the EMD. The application is built independently from the existing EMD and can be connected to any application programming interface that provides the related EMD data. The dashboard offers a “front end” summary to be linked to the EMD systems and online parent portal. The final version of the visualization design is tested and operational in the browsers used in the specific context (the CHC organizations uses Chrome and Firefox).

### Prototype and Downstream Validation

So far, the described procedure resulted in a comprehensible 360° CHILD-profile, usable on computer and mobile devices (laptop or tablet) and printable for home visits. This visualization of CHC data at a glance is validated on impact at the level of algorithm design and visual encoding and interaction design and is ready to be introduced to CHC practice. Field study with focus on downstream validation on the level of operation and data type abstraction is beyond the scope of this article. This field study will be separately presented in feasibility RCT’s protocol and result papers on this study which includes quantitative and qualitative research.

## Discussion

### Overview

This paper describes the stepwise development of a new dashboard, which combines visualization and theoretical ordering of health data based on the ICF-CY framework, to offer guidance on how to use the nested design model to achieve visualization of a comprehensive overview at a glance.

In this example, the practical implementation of the ICF-CY framework to summarize electronic health records is intended to display coherence between different health domains. The goal is to facilitate analytic thought processes during shared decision making toward preventive, individualized health plans directed at promoting health [26,32].

The CHILD-profile is designed to optimally display a holistic overview of data from electronic health records in line with the ICF-CY framework and enables considering multiple perspectives on child’s development and health. Within the ongoing project, the dashboard itself was evaluated while taking into account several perspectives.

### Strengths and Limitations

This project shows us which opportunities can arise from bringing together expertise/experience from the medical and information visualization/human–computer interaction field of knowledge. This collaboration, not yet common within health care, leads to synergy and optimal ground for realizing meaningful visualization of complex health information and sufficient adoption rate and essential performance in practice.

Additionally, the choice for a user-centered design approach, with active involvement of relevant stakeholders in every design level, increases the likelihood of usability within CHC practice and reaching the intended goals [17].

The currently experienced problems with EMD concerning accessibility of health data are avoided in this new information technology by considering international standards of human–computer interaction for information representation (ISO 9241-12 [25]) as well as theoretical aspects of design based on prior research in this field [26,27].

The nested design model is especially suitable for the context of data visualization within health care as it offers a holistic perspective on the design process [15]. For each level of design, evaluation during development (upstream validation) and after finishing the data-visualization design (downstream validation) is included. By integrating these design and evaluation methods, knowledge is generated on how to deliver a solid visualization with performance as intended as well as on how to measure actual effectiveness in practice and interpret the findings during implementation. However, it is important to note that the nested design model offers researchers a framework for structuring the design process on a rather abstract level. For each specific visualization, the choice for design and evaluation methods and the operationalization should be customized to the content and aim of the visualization and the context in which it will be implemented.

As we can only understand how people use a new tool when it exists, we could only partly tackle downstream validation within this project. Early versions of the dashboard and prototype are technically tested and qualitative tests are performed with rather limited study populations. To complete downstream validation process, studies with higher numbers of participants must be performed to reach sufficient power to evaluate if the innovation contributes to experienced needs in practice and leads to the intended health outcomes.

### Opportunities and Challenges

By utilizing the ICF-CY as a framework for ordering health data, professionals are provided with an interactional structure for aggregating details of an individual’s unique health reality across several dimensions. This structure makes it not only possible to comprehensibly display the multidimensionality of health but also the coherence between different health domains. Therefore, we hypothesize that the use of this new dashboard in CHC practice can:

- support to identify strengths, challenges, needs, and goals and “entry points” for health management;
- automatically guide (mostly subconsciously) “thinking processes” toward a more predictive, personalized, and participative approach of health;
- improve health literacy and facilitate shared decision making.

The modern information technologies, used to deliver a functional profile, allow greater direct access to health information for parents and youth (during visits and at home via online portal). By providing parents/youth insight into health facilitators and barriers, we think they will be empowered to take a more proactive, leading role during decision-making processes and make preventive health plans fit their context.

To study usability, adoption rate, and performance (regarding the intended goals) in practice, a field study and other follow-up studies need to be performed with sufficient power. To complete the validation process, it is important to measure ordering effects, visual salience, and bias effects, considering variables such as educational background and others. It is, however, a challenge to perform effect studies with sufficient sample sizes within the multidisciplinary and heterogeneous context of the preventive CHC. Therefore, the first study to be performed will be a pragmatic feasibility RCT, in which both CHILD-profile’s feasibility and RCT’s feasibility aims will be evaluated. The RCT protocol and results will be published in separate articles [31]. Results of this field study will offer underpinning of necessary requirements for successful follow-up effect studies with sufficient power.

After completion of downstream validation and effective implementation of this new tool in CHC, we anticipate that using the CHILD-profile within CHC will stimulate toward more complete and uniform data registrations. This would lead to availability of standardized and theoretically structured health data (in accordance with the ICF-CY framework), which are more fit for epidemiological research and future possibilities like automatic transformation toward internationally standardized ICF codes.

### Conclusions

This work is an important step toward bridging the information asymmetry between electronic health data, physicians, and patients and clients in general.

We propose the nested design model as a method to structure the design process while considering validation cycles for each level of design, both immediately during the process and impact-oriented validation after implementation, considering the effects of individual aspects on performance in practice.

We provide guidance on how to utilize the design model in a health context based on a concrete example and specific guidelines on how to address heterogeneous capabilities within preventive CHC through visual means and interaction design.

In our design study we developed a working prototype of a comprehensible 360° CHILD-profile on which CHC data are visualized at a glance. The application automatically converts CHC health data, already registered in the EMD, into a visualization which represents the continuum-based context of children’s health and development.

## Appendix

Multimedia Appendix 1 An example of schematic description of a professional within CHC-context (an integration of empathy map and persona).

## Abbreviations

BPS: Bio-Psycho-Social model of health
CHC: preventive Child Health Care
CSS: Cascading Style Sheets
DSM-5: Diagnostic and Statistical Manual of Mental Disorders, 5th edition
EMD: electronic medical dossier
ICD-11: International Statistical Classification of Diseases and Related Health Problems, 11th revision
ICF-CY: International Classification of Functioning, Disability and Health: Children and Youth version
ISO: International Organization for Standardization
MD: medical doctor
PHC: personalized health care
RCT: randomized controlled trial
