# Supplementary material for: Designing a Personalized Health Dashboard: Interdisciplinary and Participatory Approach
Source: JMIR Form Res. 2021 Feb 9;5(2):e24061. doi: 10.2196/24061 (PMC7902185; doi:10.2196/24061)
Supplement: Multimedia Appendix 1 [file formative_v5i2e24061_app1.pdf]

Nurse

in

Child Health Care

Education level:
Higher professional (nurse)
Personality:
- Social, concerned
- Dutiful, executive

Consultations

Goal
- Explore health situation
- If needed, refer to CHC-medical doctor or other caregivers

Work activities
Mostly regular consultations
Some consultations on indication

- 8 - 10 children during one morning
- 20 - 30 minutes per consultation

Competencies
- Ability to assess the health situation
- Neutral and open communication
- Give appropriate attention to parent and child

1) Preparation: study EMD
2) Start conversation: introduction
3) Talk over checklist conform EMD
4) Go over questionnaire
5) Measurement of length and weight etc.
6) Conclusion + decision making

Gains

Overall wishes and needs
- A clear overview of results/conclusions
- To quickly gain an overall view on how the child is doing
- Checklist of topics of conversation
- Active involvement of parents

"If you mark a signal item, it will show in the start screen"

Wishes and needs concerning the 360°CHILD-profile:
- To provide a clear overview of health data collected
- To provide the right signals
- The possibility to show parents cohesion between health domains
- To add several data:

  "The pro-social factors, the reciprocity is often forgotten"
  "You cannot retrace the performed tests"
  "A conclusion from every item"

"If I find a signal item relevant enough to re-read, I must be able to quickly find it back in the EMD"

Pains

The EMD
- The Electronic Medical Dossier the CHC-nurses currently work with, hinders their work activities/ tasks.

"If signal items are not marked, relevant data are hard to find"

"It costs time to retrace information about medical history, as previous registrations must be opened""

Time pressure
- The CHC-nurse is bound to the limited timeframe per consultation.

"But I also have to type all registrations in the EMD"
